# Antibacterial activities and toxicological study of the aqueous extract from leaves of *Alchornea cordifolia* (Euphorbiaceae)

**DOI:** 10.1186/s12906-017-1854-5

**Published:** 2017-07-04

**Authors:** Merline Namekong Djimeli, Siméon Pierre Chegaing Fodouop, Guy Sedar Singor Njateng, Charles Fokunang, Donald Sedric Tala, Fabrice Kengni, Donatien Gatsing

**Affiliations:** 1 Department of Biochemistry, Faculty of Science, University of Dschang, P.O. Box 67, Dschang, Cameroon; 2 Department of Biomedical Sciences, Faculty of Science, University of Ngaoundéré, P.O. Box 454, Ngaoundéré, Cameroon; 3 Department of pharmacotoxicology and pharmacokinetics, Faculty of Medicine and Biomedical Sciences, University of Yaoundé I, P.O. Box 337, Yaoundé, Cameroon

**Keywords:** *Alchornea Cordifolia*, *E. coli*, Toxicity, Mice

## Abstract

**Background:**

*A. cordifolia* is a plant widely used in Africa to solve many health problems. In Cameroon, it is used in the treatment of urogenital infections. As a continuation of our search for pharmacologically active agents from natural sources, the antimicrobial activity of *A. cordifolia* leaf extracts against *E. coli* and the toxicity of this extract were investigated.

**Methods:**

The antibacterial activity of the aqueous extract from leaves of *Alchornea cordifolia* was carried out in vitro on *Escherichia coli*, as well as in vivo on *E. coli*-infected rat model. Phytochemical screening was performed using standard methods. The acute toxicity was investigated in mice, while at the end of treatment of infected rats, some biochemical, hematological and histological markers of toxicity were evaluated.

**Results:**

The extract exhibited a bacteriostatic activity with MIC value of 1500 μg/ml. Phytochemical screening revealed the presence of phenols, tannins, triterpens, flavonoids, alkaloids, anthraquinones, anthocyanins, saponins and coumarins in the extract. The acute toxicity study showed LD_50_ values of 8.6 g/kg and 3.8 g/kg in male and female mice respectively. In vivo, the oral administration of the extract showed a dose-dependent decrease of the bacterial load as the extract at 232, 112 and 58 g/kg were able to eradicate the infection after 9, 11 and 13 days of treatment. The infected rats showed a significant (*p* < 0.05) increase in the level of serum creatinine, ALAT, white blood cells, and a significant (*p* < 0.05) decrease in the level of food and water intake, the relative weight of lungs, heart and spleen. In the treated rats, a significant (*p* < 0.05) increase in food and water intake and ALAT was observed at the doses of 116 and 232 mg/kg. A decrease in the red blood cells count and serum protein levels was also observed. These observations corroborate liver damages as revealed by the histopathological examination of the cross sections of this organ.

**Conclusion:**

The results of this assay thus showed that the extract of *A. cordifolia* is bacteriostatic, therapeutic at 58 g/kg bw and may be considered as slightly and almost non-toxic on females and males mice respectively.

## Background

*A. cordifolia* is a plant widely used in Africa alone or in association with other plants to solve many health problems [1]. The leaves are simple and alternate, and are heart shaped at the base with long petiole. The inflorescence consists of auxiliary panicles and the flowers are greenish white [1]. In Cameroon, this plant is very used in the treatment of urogenital infections sometimes caused by human commensalist bacteria, which become pathogen either due to a change in their normal behaviour/habitat or to a failure in the immune system [2]. Some of these bacteria are *E. coli*, *P. mirabilis*, *S. aureus*, *K. pneumoniae* and *P. Aeruginosa.* When neglected, these infections cause remarkable consequences such as prostatite, nephrite, sterility and can in some cases increase cancer risks [3]. Thus, there is a need to find means to combat these infections. Natural substances among which those from medicinal plants such as *A. cordifolia* may constitute a good source of theurapeutics that can be used to fight efficiently against these diseases. Previous investigations on *A. cordifolia* showed that its leaf ethanol extract was able to delay mouse intestinal transit accelerated by castor oil, inhibit the production of diarrhoeal faeces and modify the fluid and electrolyte transport across the colonic mucosa when administered intraluminally [4]. The methanolic extract of its leaves showed antipyretic [5], hepatoprotective as well as antioxidant activities [6]. Furthermore, the aqueous ethanolic extract from *A. cordifolia* leaf was shown to be capable of inducing elastogenesis in the aorta [7]. However, to the best of our knowledge, no information on its aqueous extract activityagainst *E. coli* as far as its toxicological profile is available. As a continuation of our search for pharmacologically active agents from natural sources with potential for the treatment of urogenital infections [8], the antimicrobial activity of *A. cordifolia* leaf extracts against *E. coli* which represent about 75 to 95% of cases of these infections [9], and the toxicity of this extract were investigated.

## Methods

### Plant material

The leaves of *A. cordifolia* were collected from Dschang locality, Menoua division, West region in April 2010. Authentification was carried out by the botanists of the Cameroon National Herbarium, Yaounde, where a voucher specimen (9656/SRF CAM) was deposited.

### Test bacteria and culture media

The test microorganism, *Escherichia coli,* was obtained from the Medical Bacteriology Laboratory of the Pasteur Center, Yaounde, Cameroon. *Escherichia coli* ATCC 10536 obtained from American Type Culture Collection was used as reference strain. Three types of culture media were used during the work: Mueller Hinton Agar (MHA) for the determination of the minimum bactericidal concentration, Mueller Hinton Broth (MHB) for the determination of the minimum inhibitory concentration and Mac conkey agar (MCA) for *E. coli* culture.

### Experimental animals

In the present study, 60 *Swiss* albino mice (30 males and 30 females) weighing 18–24 g, and 35 *Wistar* albino female rats weighing 148–187 g were used. These animals were bred in the animal house of the University of Dschang, Cameroon.

### Preparation of the extract

The leaves of *A. cordifolia* were allowed to dry at room temperature (24 ± 2 °C) and were ground. 100 g of the powder were macerated at room temperature in 1 l of water for 48 h and filtered with n^o^ 1 whatman paper. The filtrate was concentrated in a drying oven at 45 °C to obtain12.64 g of crude extract [8].

### Antibacterial test

The in vitro antibacterial activity of the extract was performed by determining the minimum inhibitory concentrations using broth microdilution method [8]. Briefly, bacterial suspensions of about 1.5 × 10^8^ CFU/ml (Mc Farland turbidity standard no. 0.5) were prepared. To obtain the inocula, these suspensions were diluted 100 times in Muller Hinton broth to give 1.5 × 10^6^ CFU/ml. The antimicrobial susceptibility tests were performed in 96 wells microplates. A serial two-fold dilution of the plant extract was performed to obtain final concentration range of 6000 to 46.87 μg/ml for the extract and of 20 to 0.157 μg/ml for the ciprofloxacin which served as reference drug (positive control) in a total volume of 200 μl/well. Each well contained the test substances at a particular concentration and the bacterial suspension (100 μl) in Muller Hinton broth. For every experiment, sterility control (5% *v*/v aqueous DMSO and broth) and negative control made up of 5% *v*/v aqueous DMSO, broth and inoculum were included. The plate was later covered with sterile cover and incubated at 37 °C for 24 h. Growth was monitored colorimetrically using p-iodonitrotetrazolium violet (INT). Viable bacteria change the yellow dye of p-iodonitrotetrazolium violet to a pink colour. All concentrations at which no visible colour changes were observed were considered as inhibitory concentrations and the lowest of these concentrations was considered as the MIC.

The ridges (zigzag) method was used to determine minimal bactericidal concentrations (MBCs). In fact, the wells which showed no growth were cultivated on already prepared petri dishes of 90 mm containing MHA. After 24 h of incubation at 37 °C, concentrations which presented no bacterial growth were considered as bactericidal and the lowest was the MBC.

### Phytochemical screening

The phytochemical screening was performed qualitatively using standard methods [10]. The plant sample was screened for the following classes of compounds: phenols, tannins, terpenoids, flavonoids, steroids, alkaloids, anthraquinones, anthocyanins, saponins, coumarins.

### Acute toxicity

In acute toxicity studies, 60 Swiss albino mice (30 males and 30 females) were used. Animal treatment was performed according to the method previously described [11]. 3 h following administration of the test substance, the animals were observed frequently for bihavioural and observable physiological variations. The death animals were counted within the first 48 h and the lethal dose 50 (LD50) was determined [12]. Surviving animals were further observed for 2 weeks, during which their weight, food and water intake were recorded.

### Infection and treatment

In vivo antibacterial and toxicity studies were performed according to the method previously described [11] with some modifications. The animals were divided into six groups (5 males and 5 females): one simple control (animals who received distilled water), one negative control (infected but not treated), one positive control (infected animals that received the standard antibiotic) and three extract treated groups (58 mg/kg, 116 mg/kg and 232 mg/kg) based on the tradipratician dose which was 58 mg/kg. All the animals were immunosuppressed by intraperitoneal administration of cyclophosphamid at 30 mg/kg, and the infection was done on the third day by intravaginal administration of 100 μl *E. coli* (1.2 × 10^8^ CFU/ml). From the fourth day post-infection, animals were daily treated with the *A. cordifolia* extract. The administration of various doses of extract, antibiotic and distilled water was done by gastric intubations once a day, for two consecutive weeks. The food and water intakes were evaluated every day and the animals were also weighed. The evolution of the bacterial load as a function of time was determined based on the diluted vaginal sample.

### Effect of the *Alchornea cordifolia* extract on haematological, biochemical and histological parameters

At the end of treatment, the animals were anesthetized with chloroform vapour prior to dissection. Blood samples were collected by cardiac puncture into heparinised and non- heparinised centrifuge tubes. The heparinised blood was used to estimate hematocrit values, while the non-heparinised blood was allowed to coagulate, centrifuged and the serum was separated. Serum was assayed for proteins, cholesterol, triglycerides, creatinine, and Transaminases (ALT and AST). Immediately after blood collection, the animals were killed for tissue study. Liver, lungs, heart, kidneys, and spleen were isolated, and weighed. Part of each of these organs was cut and stored at −30 °C for the determination of protein concentration, while pieces of livers were kept for the histological analyses.

#### Preparation of serum sample

The blood was allowed to clot by standing at room temperature for 1 h and then refrigerated for another 1 h. The resultant liquid part was removed and centrifuged at 3000 xg for 5 min, and then the serum (supernatant) was obtained and stored at −30 °C for analysis.

#### Preparation of tissue homogenate

The homogenate of each organ was prepared in 0.9% NaCl solution at the concentration of 15% (i.e. 15 g organ in 100 ml of solution). Possible damages to the liver, kidneys, heart, lungs, spleen, and red blood cells of the animals as a result of repeated administration of the *Alchornea cordifolia* extract was studied using some biochemical parameters of tissue damages. Total protein concentrations of the above-mentioned organs were determined by the Biuret method [13]. Serum cholesterol, triglycerides and creatinine levels were determined by colorimetric method using commercial kits of INMESCO [14]. Serum transaminases (ALT and AST) activities were determined by the kinetic method [15, 16] using commercial kits of INMESCO. The determination of the hematological parameters [17] and the histological study were perfomed [18].

### Statistical analysis

Statistical analyses were performed using SPSS for Window software version 12.0. Results were expressed as mean ± standard deviation using the ANOVA and the means of different groups were compared using the Waller-Duncan test where *P* value less than 0.05 was considered statistically significant.

## Results

### Antibacterial activity and phytochemical screening

The in vitro study showed a MIC value of 1500 μg/ml and a MBC value greater than 6000 μg/ml for the isolate. This later was less sensitive compared to the reference strain (MIC = 750 μg/ml). The ratio MBC/MIC was more than 4 indicating that this extract possesses a bacteriostatic activity.

Phytochemical screening of the extract revealed the presence of different classes of chemical compounds, namely phenols, tannins, terpenoids, flavonoids, steroids, alkaloids, anthraquinones, anthocyanins, saponins and coumarins except steroids (Table 1).

**Table 1:** Chemical groups of compounds found in the aqueous extract from the leaves of *A. cordifolia*

Active compounds	State
Alkaloids	+
Anthocyanins	+
Anthraquinones	+
Coumarins	+
Flavonoids	+
Phenols	+
Saponins	+
Steroids	−
Tannins	+
Terpenoids	+

-: absent; +: present

### Acute toxicity

The behavioural changes of animals observed during acute treatment with the extract are summarized in Table 2. The mice were observed for activity (locomotion), reaction to noise, reaction to pinch, aggressiveness, state of excrement, and for mortality (within 48 h) after administration of the various doses of water macerated extract of *A cordifolia*. In both sexes, locomotion, reaction to noise and pinch were reduced in a dose-dependent manner. In both sexes, the excrement was granular at 2 g/kg and 4 g/kg, doughy at 4 g/kg and 8 g/kg respectively, and liquid at 32 g/ kg. Fourteen cases of death were recorded for males and 19 for females within 48 h after administration of extract. The lethal dose 50 (LD_50_) in male mice was calculated to be 8.6 g/kg, while in females it was 3.8 g/kg.

**Table 2:** Behavioural changes observed during the acute toxicity study of the aqueous extract from *A. cordifolia*

Studied parameters	Doses (g/kg)											
	Males						Females					
	0	2	4	8	16	32	0	2	4	8	16	32
Locomotion	N	N	N	D	D+	D+	N	N	D	D+	D+	D+
Reaction to noises	N	N	D	D+	D+	D+	N	N	N	D	D+	D+
Reaction to pinch	N	N	N	D	D+	D+	N	N	N	N	D	D
State of the tail	N	N	N	N	N	N	N	N	N	N	N	N
State of excrements	G	G	P	P	P	L	G	G	G	P	P	L
Aggressiveness	N	N	N	N	N	N	N	N	N	N	N	N
Mortality within 48 h	0	1	1	2	5	5	0	1	3	5	5	5

*N* Normal, *D* decreased, D+: Profoundly decreased; *G* Granular, *P* Doughy, *L* liquid

**Fig. 1:**
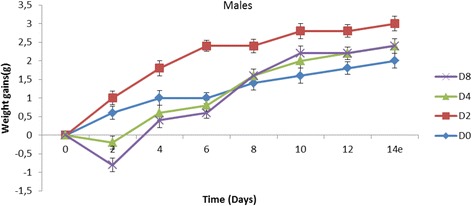
Trends in mean body weight gains of male mice after single dose oral administration of *A. cordifolia* leaves extract. D0 = distilled water, D2 = 2 g/kg, D4 = 4 g/kg, D8 = 8 g/kg

**Fig. 2:**
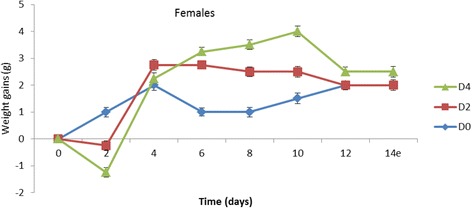
Trends in mean body weight gains of female mice after single dose oral administration of *A. cordifolia* leaves extract. D0 = distilled water, D2 = 2 g/kg, D4 = 4 g/kg

From the Figs. 1 and 2 that present the effect of the extract on the weight of the animals, it can be observed that, at 2 g/kg, the weight of males increased while that for females decreased. At 4 g/kg, a decrease in the animal weight was observed in both sexes. The same observation was made at 8 g/kg at which all the females died.

An increase in water and food intake in both sexes depending on the dose administered was also observed (Tables 3 and 4). However, there was a decrease in water intake in males at the first week before an increase in the second week. Generally, the extract caused an increase in food intake at the second week of experiment. This increase is significant (*p* < 0.05) for females that received the dose of 4 g/kg and for males that received the dose of 8 g/kg. However, there was a decrease in food intake in the first week at the doses of 8 and 4 g/kg for male and females respectively.

**Table 3:** Evolution of water consumptions of rats after single administration of the aqueous extract from *A. cordifolia*

		Water Intake (ml/week)	
Sex	Dose (g/kg)	Week1	Week2
Males	0	34.18 ± 2.95^a^	34.50 ± 2.25^b^
	2	30.02 ± 2.54^b^	42.12 ± 3.21^a^
	4	32.60 ± 0.95^b^	44.76 ± 1.97^a^
	8	32.82 ± 0.54 ^b^	45.00 ± 0.19^a^
Females	0	38.26 ± 3.25^b^	44.26 ± 2.55^b^
	2	42.26 ± 1.90^ab^	45.50 ± 2.13^b^
	4	45.12 ± 2.12^a^	50.10 ± 1.02^a^

Tabulated values are mean ± SEM of five trials. Values of the same column with different letters are significantly different at *P* < 0.05

**Table 4:** Evolution of food consumptions of rats after single administration of the aqueous extract from *A. cordifolia*

	Food Intake (g/week)		
Sex	Dose (g/kg)	week 1	Week 2
Males	0	58.10 ± 2.12^a^	56.14 ± 2.03^b^
	2	56.05 ± 3.99^a^	57.26 ± 1.18^b^
	4	53.34 ± 2.21^ab^	58.12 ± 0.31^b^
	8	48.26 ± 2.15^b^	75.00 ± 1.32^a^
Females	0	53.62 ± 1.13^a^	54.36 ± 2.00^b^
	2	45.76 ± 4.33^ab^	55.50 ± 2.45^b^
	4	40.75 ± 3.98^b^	62.02 ± 4.90^a^

Tabulated values are mean ± SEM of five trials. Values of the same column with different letters are significantly different at *P* < 0.05

### Infection and treatment of animals

#### Food and water intakes

From the Table 5 that shows values of water and food intake, it can be observed a significant increase (*p* < 0.05) in food intake for rats that received the doses of 116 and 232 mg/kg during the first week of treatment. In the second week, the food intake of the rats was comparable to that of that normal control. On the contrary, a significant decrease (*p* < 0.05) in food intake in infected but not treated rats during the second week was observed. During the first week, the water intake of the animals was similar to that of normal control, except for those treated with the dose 232 mg/kg which had significantly increased (*p* < 0.05). In the second week, the water intake of all the treated animals was similar to that of the normal control; while in the infected but non treated control, a significant decrease (*p* < 0.05) in water intake was observed.

**Table 5:** Food and water intakes of rats as affected by doses of *A. cordifolia* leaves extract during treatment

Doses	Food Intake (g/week)		Water Intake (ml/week)	
	Week 1	Week 2	Week 1	Week 2
0- mg/kg	24.00 ± 1.58^b^	24.60 ± 2.30^b^	15.60 ± 2.61^bc^	18.60 ± 1.14^a^
0i mg/kg	18.60 ± 1.14^c^	20.80 ± 2.59^c^	14.00 ± 2.00^c^	14.60 ± 2.28^b^
58 mg/kg	24.90 ± 2.64^ab^	26.20 ± 1.48^ab^	16.80 ± 3.27^abc^	16.60 ± 1.70^ab^
116 mg/kg	26.00 ± 2.91^a^	27.00 ± 2.47^ab^	18.40 ± 3.07^ab^	18.00 ± 1.92^a^
232 mg/kg	27.60 ± 2.70^a^	29.00 ± 2.70^a^	25.60 ± 2.70^a^	19.20 ± 1.71^a^

0i: Infected and non-treated, O-: Normal control. Tabulated values are mean ± SEM of five trials. Values of the same column with different letters are significantly different at *P* < 0.05

#### Weight of the rats

There was no significant difference (*p* < 0.05) in the weight of all the animals in the first week. In the second week, we observed a significant increase in infected animals treated with 116 and 232 mg/kg, while there was a decrease in weight for those infected but not treated (Table 6).

**Table 6:** Trends in mean body weight gains of rats during 2 weeks of treatment

Mean body weight gains (g/week)		
Doses	Week 1	Week 2
0- mg/kg	154.40 ± 7.78^a^	163.43 ± 5.51^b^
0i mg/kg	153.23 ± 6.19^a^	159.89 ± 6.04^c^
58 mg/kg	155.86 ± 8.02^a^	169.18 ± 6.17^b^
116 mg/kg	158.98 ± 5.76^a^	175.71 ± 5.39^a^
232 mg/kg	157.56 ± 6.77^a^	174.71 ± 7.23^a^

0i: infected and non-treated, O-: normal control. Tabulated values are mean ± SEM of five trials. Values of the same column with different letters are significantly different at *P* < 0.05

#### Evolution of the bacteria load as a function of time

**Fig. 3:**
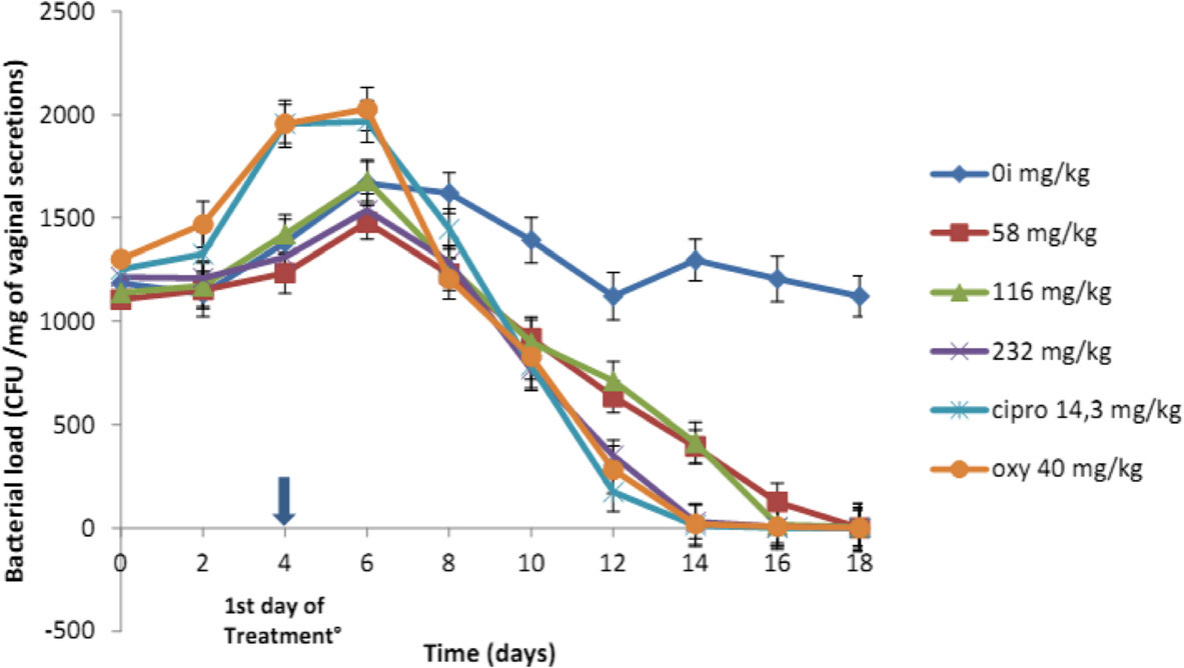
Evolution of the bacterial load as a function of time

The Fig. 3 shows that except for the group that receives ciprofloxacin, the bacteria load increase in all the groups from infection up to the first 2 days of treatment; while it decreases for all the treated animals that received the dose 232 mg/kg, oxytetracyclin and ciprofloxacin. At the 10th day of treatment, there was a total absence of colonies in the animals treated with the dose 232 mg/kg; this absence was observed for those treated with the doses 116 and 58 mg/kg at the 12th and 14th day respectively. For the infected and non-treated control, we observed a decrease in the number of colonies up to the 8th day, an increase from the 8th to the 10th day, and then a decrease. This decrease is not continuous but tends to remain around a fixed value of 1300 CFU/ml on the 14th day post-infection.

#### Effect of water macerated leaves of *A. cordifolia* on biochemical parameters

In all the animals, even in those infected, there was no effect in the relative weight of the kidney and liver (Table 7). However, a significant decrease (*P* < 0.05) in relative weight of spleen, lung, and heart when compared with the normal control was observed. This decrease is intense in the infected and non-treated animals and those treated with the dose of 58 mg/kg.

**Table 7:** Organ to body weight ratios as affected by infection and doses of *A. cordifolia* extract after 2 weeks of administration to rats

Relative weight (g/100 g of body weight)					
Doses	Spleen	Lungs	Heart	Liver	Kidneys
0-mg/kg	0.56 ± 0.14^a^	1.21 ± 0.35^a^	0.40 ± 0.04^a^	3.85 ± 0.42^a^	1.19 ± 0.30^a^
0i mg/kg	0.32 ± 0.04^c^	0.82 ± 0.16^b^	0.32 ± 0.03^b^	3.72 ± 0.45^a^	0.98 ± 0.08^a^
58 mg/kg	0.38 ± 0.05^bc^	0.83 ± 0.08^b^	0.32 ± 0.01^b^	3.49 ± 0.32^a^	1.03 ± 0.05^a^
116 mg/kg	0.42 ± 0.04^bc^	0.85 ± 0.08^b^	0.33 ± 0.03^b^	3.34 ± 0.23^a^	1.00 ± 0.15^a^
232 mg/kg	0.44 ± 0.04^b^	0.87 ± 0.16^b^	0.36 ± 0.02^ab^	3.57 ± 0.35^a^	1.04 ± 0.16^a^

0i: Infected and non-treated control, 0-: Normal control. Tabulated values are mean ± SEM of five trials. Values of the same column with different letters are significantly different at *P* < 0.05

The serum and urine creatinine of the rats are comparable to those of the normal control (Table 8). However, a significant increase (*P* < 0.05) in the level of serum creatinine in the infected and non-treated rats was observed. There is no significant difference (*p* < 0.05) between total cholesterol, HDL cholesterol, triglycerides and atherosclerosis index in all groups when compared with the control counterpart (Table 8). There was no significant difference (*P* < 0.05) in the amount of proteins present in the liver, the heart, kidneys and the spleen in all the groups when compared with the normal control (Table 8). On the contrary, at the level of the lungs, a significant decrease (*p* < 0.05) in proteins for the animals when compared with the normal control was observed. A decrease in the amount of serum protein was also observed for the rat treated with the *A. cordifolia* extract (Table 8). The results of the effect of *A. cordifolia* on Transaminases shows that for animals treated with dose 58 mg/kg, the level of Alanine Transaminase (ALAT) was similar to that of the normal control (Table 8). There was a significant increase (*p* < 0.05) in the infected but non treated control and those treated with the dose 116 as well as 232 mg/kg. In the case of Aspastate Transaminase (ASAT) there was a significant decrease (*p* < 0.05) in the rats treated with 58 mg/kg, while for those treated with 116 mg/kg, 232 mg/kg and those infected but non-treated, it was similar to the normal control (Table 8).

**Table 8:** Effects of doses of aqueous extract from *A.cordifolia* on biochemical parameters after 2 weeks of administration to rats

Studied Parameters	Doses				
	0- mg/kg	0i mg/kg	58 mg/kg	116 mg/kg	232 mg/kg
Serum Creatinine (mg/ dl)	2.64 ± 0.19^b^	5.00 ± 0.92^a^	2.31 ± 0.88^b^	2.62 ± 1.15^b^	2.65 ± 0.97^b^
Urine Creatinine (mg/ dl)	25.35 ± 3.88^a^	26.88 ± 2.28^a^	25.85 ± 5.15^a^	26.38 ± 3.64^a^	28.09 ± 3.45^a^
Total Cholesterol (mg/ dl)	288.89 ± 14.43^a^	285.26 ± 11.47^a^	329.38 ± 13.53^a^	335.90 ± 12.31^a^	340.72 ± 5.16^a^
HDL Cholesterol (mg/dl)	58.60 ± 3.30^a^	51.91 ± 4.36^a^	65.21 ± 5.97^a^	63.67 ± 3.87^a^	62.50 ± 2.68^a^
Atherosclerosis Index(mg/ dl)	3.98 ± 0.86^a^	4.58 ± 0.55^a^	4.51 ± 0.12^a^	4.78 ± 0.74^a^	4.55 ± 1.07^a^
Triglycerides (mg/dl)	114.85 ± 7.09^a^	102.46 ± 10.65^a^	110.10 ± 7.92^a^	119.64 ± 6.44^a^	126.29 ± 5.94^a^
Renal protein (mg/g)	3.81 ± 0.95^a^	3.67 ± 1.11^a^	3.72 ± 1.07^a^	3.98 ± 1.02^a^	4.02 ± 0.29^a^
Spleen protein (mg/g)	72.73 ± 9.71^a^	71.82 ± 8.40^a^	75.55 ± 6.79^a^	79.64 ± 9.58^a^	84.36 ± 7.82^a^
Pulmonary protein (mg/g)	59.09 ± 3.02^a^	42.81 ± 2.22^b^	40.97 ± 4.26^b^	41.58 ± 4.21^b^	45.36 ± 3.86^b^
Hepatic protein (mg/g)	148.73 ± 2.70^ab^	127.91 ± 12.18^b^	134.82 ± 5.94^b^	141.82 ± 6.31^ab^	154.91 ± 13.08^a^
Cardiac protein (mg/g)	53.94 ± 5.08a	40.03 ± 2.57b	42.73 ± 2.49ab	46.00 ± 6.08ab	49.45 ± 4.71ab
Serum protein (mg/g)	413.36 ± 19.7a	410.57 ± 12.60a	395.39 ± 14.57b	400.02 ± 17.01b	402.91 ± 10.58b
Urine protein (mg/g)	3.64 ± 0.94^a^	3.24 ± 0.20^ab^	2.80 ± 0.14^b^	3.28 ± 0.21^ab^	3.15 ± 0.32^ab^
ALAT (UI/l)	30.36 ± 2.92^b^	39.42 ± 3.51^a^	34.86 ± 1.74^ab^	41.92 ± 2.33^a^	43.79 ± 2.44^a^
ASAT (UI/l)	55.19 ± 3.18^a^	51.90 ± 4.21^ab^	49.02 ± 3.49^b^	53.88 ± 4.34^ab^	57.11 ± 4.33^a^

0-: Normal control, 0i: Infected and non-treated. Tabulated values are mean ± SEM of five trials. Values of the same row with different letters are significantly different at *P* < 0.05

As far as hematological parameters were conserned, no significant difference (*p* < 0.05) between the number of white blood cells, the percentage of hematocrit and the hemoglobin in all the groups when compared with the normal control was observed (Table 9). However a significant increase (*p* < 0.05) in the level of white blood cell in the infected but non treated rats was observed. Also, a significant decrease (*p* < 0.05) in the level of red blood cells in the animals that received the doses 116 and 232 mg/kg was observed (Table 9).

**Table 9:** Effects of doses of water macerated leaves extract of *A*.*cordifolia* on hematocrit, red blood cells, white blood cells and hemoglobin values after 2 weeks of administration to rats

Doses	Hematocrit (%)	Red blood cell (×10^6^ /mm ^3^ de sang)	White blood cell (×10^6^ /mm ^3^ de sang)	Hemoglobin (g/dl)
0- mg/kg	39.00 ± 2.45^ab^	3.46 ± 0.97^b^	8.04 ± 1.42^a^	16.36 ± 2.46^ab^
0i mg/kg	41.35 ± 1.32^a^	12.77 ± 1.40^a^	7.42 ± 0.97^a^	20.43 ± 2.59^a^
58 mg/kg	40.03 ± 1.23^a^	3.28 ± 1.73^b^	7.05 ± 0.53^ab^	15.48 ± 0.70^ab^
116 mg/kg	38.98 ± 2.02^ab^	2.90 ± 1.68^b^	6.85 ± 0.37^b^	15.26 ± 0.51^ab^
232 mg/kg	35.84 ± 3.67^b^	5.06 ± 0.28^b^	6.78 ± 0.36^b^	13.17 ± 1.06^b^

0i: Infected and non-treated, 0-: Normal control. Tabulated values are mean ± SEM of five trials. Values of the same column with different letters are significantly different at *P* < 0.05

### Histology

**Fig. 4:**
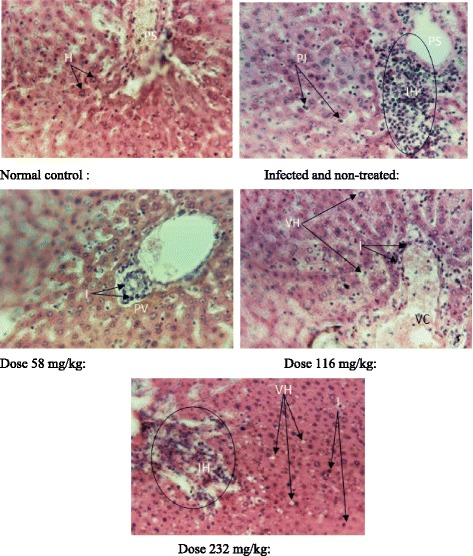
Liver histopathological analysis. H: Hepatocytes, PI: Parenchyma inflammation, PS: Portal space, IH: Inflammation hall, I: Light inflammations, PV: Portal vein, VH: Vacuolization of hepatocytes, VC: Small vascular congestion

The histological study shows that the liver was normal for the normal control. However, there were inflammations in the infected and non-treated rats and those infected and treated with the extract. At 116 mg/kg, a vascular congestion was noted. Furthermore, a dose-dependent vacuolisation of the hepatocytes at the doses of 116 mg/kg and 232 mg/kg was observed (Fig. 4).

## Discussion

### Phytochemical screening and antibacterial activities

The leaves extract from *A. cordifolia* presented antibacterial activity against *Escherichia coli* with a MIC value of 1500 μg/ml. this may be ascribed to the different classes of compounds found in the extract. In fact, the phytochemical screening of this extract revealed the presence of phenols, tannins, terpenoids, flavonoids, steroids, alkaloids, anthraquinones, anthocyanins, saponins and coumarins. Individual activities of these compounds have been demonstrated [19].

### Acute toxicity

Some side effects were observed in both sexes: the decrease in sensibility to stimuli (pinch and noise), the decrease in the mobility and the softness of the feces. The decrease of the sensibility to pinch in both sexes could be due to the effect of the extract on the perception of the pain by some nocireceptors, or by the inhibition of algogenic substances (i.e. prostaglandin, histamin), or also by the inhibition of pain transmission at the central level [20]. In fact, some drugs prevent the perception of pain (analgesic) by inhibiting the conversion of arachidonic acid to prostaglandin, the substance which regulates pain perception [21]. The decrease in mobility and sensibility to noise observed in mice could be due to the sedative or depressive effect of the extract [8] or its tranquillizer effect on central nervous system and motor neurons [22]. Saponins are glycosides producing foam; they can provoke loss of appétit, apathy and weight; also gastroenteritis and diarrhea in animals [23]. Their presence in the extract may be responsible for the persistent anorexia during the first 2 days following extract administration and diarrhea that was observed some hours after the administration of the extract. The weight increased 1 week after extract administration. This could be explained by the biotransformation and elimination of the compounds responsible for the weight decrease. The LD_50_ obtained for females and males where 3.8 g/kg and 8.6 g/kg respectively. Based on Hodge and Sterner scale, this extract can be considered as slightly and almost non-toxic on females and males respectively [24].

### Infection and treatment

The decrease in food and water intake in the first week of treatment may be due to the infection which could have induced the anorexia. The increase of this consumption could be explained by the effect of the administration of the extract in rats treated with water macerated leaves of *Alchornea cordifolia*. The increase in body weight could be due to the increase in food and water intake. The increase in bacteria load before the treatment and within the first 2 days of treatment showed that bacterial infection had been established after the use of cyclophosphamid which has myelosuppressive effect [25]. The decrease in this load observed the following days could be explained by the combined action of the immune system and the extract. The decrease in lungs, heart and spleen weight could be due to the infection which may has caused the mobilization of some macromolecules toward the blood; according to the fact that at different doses of treatment there is progressive increase of these weights.

### Effect of water macerated leaves of *A. cordifolia* on biochemical parameters

Creatinine is present in little amount in the serum and in large amount in the urine. Its increase in the serum and the decrease of the urine level are signs of kidney failure [26]. The increase of the level of serum creatinine in infected but non treated rats could be due to the infection which could have caused alteration of glomerular filters and then that of kidney function. In fact, without filtration and sufficient efflux, at the level of every tubule portions, the secretion and reabsorption function are not efficient, and substances normally eliminated through the kidney remain in the blood [27]. At all doses, the creatinine level was not influenced, and then there is no effect on kidney function. The rate of cholesterol, HDL, triglycerides, and atherosclerosis index remained comparable to those of the normal control. Neither the extract nor the infection affected these parameters. In fact, an increase in the total cholesterol level could be due to the decrease of the cholesterol 7-α-hydroxylase, enzyme which catalyses the transformation of cholesterol into biliary acids [28].

Anaemia can be measured by three parameters: hematocrit, hemoglobin and the number of red blood cells [11]. The level of hemoglobin and hematocrit was similar to that of the normal control. The decrease of the number of red blood cells in rats treated by the high doses of extract could be due to the inhibition of the hematopoesis. On the other hand, the increase of the number of white blood cell observed in the infected and non-treated rats may be due to the infection. In fact, the microbial infections generally go with the stimulation of the immune system which is characterised by the increase in the level of leucocytes and immunoglobulin [29].

The increase in the amount of serum proteins and its decrease in the lungs may indicate damages at the tissue level [11]. ASAT and ALAT are considered as markers of liver function [29]. ASAT is found in the cytoplasm and mitochondria of different tissues, mainly in the heart and skeletal muscles, liver, kidneys, pancreas, and erythrocytes [30]. ALAT is an enzyme mainly present in the cytosol of hepatocytes and is then considered as a great indicator of hepathotoxicity [31]. Its rate increased at the doses 116 and 232 mg/kg; while that of ASAT remained normal. In fact, the increase in the rate of ALAT occurs when the hepatocytes are damaged [30]. This extract could then have caused damages at the liver level. These observations corroborate liver damages as revealed by the histopathological examination of the cross sections of this organ. The presence of vascular congestions on liver sections could be due to the vasoconstriction action of the *Alchornea cordifolia* extract on the walls of blood vessels [32]. The vacuolization of the hepatocytes suggests an abnormal infiltration of extracellular components in the hepatocytes, or bad functioning of the hepatocytes which do not metabolises blood nutriments normally, thus the origin of their accumulation in the cytoplasm [33]. The inflammations were observed in infected rats. These inflammations may be due to the effect of the infection for the infected but non-treated. The decreased of these inflammations in the rats that received the doses 58 and 116 mg/kg may be ascribed to the healing power of the extract. The increase of the inflammation at dose 232 mg/kg could suggest a toxicity effect of the extract at a relatively high dose. This extract could contain some substances capable of acting like non steroidal anti-inflammatory drugs by provoking a hypersensitivity reaction that led to the observed inflammation [34].

## Conclusions

The results of this assay thus showed that the extract of *A. cordifolia* possesses interesting in vitro and in vivo antibacterial activity and may be considered as slightly and almost non-toxic on females and males mice respectively.

## References

[1] Olaleye MT, Adegboye OO, Akindahunsi AA (2006). *Alchornea cordifolia* extract protects Wistar albino rats against acetaminophen-induced liver damage. Afr J Biotech.

[2] Gatsing D, Nkeugouapi CFN, Nji-Nkah BF, Kuiate JR, Tchouanguep FM (2010). Antibacterial activity, bioavailability and acute toxicity evaluation the leaf extract of *Alchornea cordifolia* (Euphorbiaceae). Int J Pharmacol.

[3] Aruoma OI (1998). Free, radicals, oxidative trace and antioxidants in human health and diseases. J Am Oil Chem Soc.

[4] Agbor GA, Léopold T, Jeanne NY (2004). The antidiarrhoeal activity of *Alchornea cordifolia* leaf extract. Phytother Res.

[5] Effo KE, Kouakou-Siransy G, Irie-Nguessan G, Sawadogo RW, Dally IL, Kamenan AB (2013). Acute toxicity and antipyretic activities of a Methanolic extract of *Alchornea cordifolia* leaves. Pharmacol Pharm.

[6] Eliakim-Ikechukwu CF, Riman EB (2009). The effect of aqueous ethanolic extract of *Alchornea cordifolia* leaf on the histology of the aorta of wistar rats. Nigerian J Physiol Sci.

[7] Osadebe OP, Okoye FBC, Uzor PF, Nnamani NR, Adiele IE, Obiano NC (2012). Phytochemical analysis, hepatoprotective and antioxidant activity of *Alchornea cordifolia* methanol leaf extract on carbon tetrachloride-induced hepatic damage in rats. Asian Pac J Tropic Med.

[8] Gatsing D, Tchakounté V, Ngamga D, Kuiate JR, Tamokou JD, Nji-Nkah BF (2009). In vitro antibacterial activity of *Crinum purpurascens* leaf extract against the *Salmonella* species causing typhoid fever and it toxicological evaluation. Iran J Med Sci.

[9] Grabe M, Bishop MC, Bjerklund-Johansen TE, Botto H, Çek M, Lobel B (2009). Urological infections. Europ Assoc Urol.

[10] Harborne JB (1973). Phytochemical methods: a guide to Mordern techniques of plant analysis.

[11] Gatsing D, Aliyu R, Kuiate JR, Garba IH, Tedongmo N, Tchouanguep FM (2005). Toxicological evaluation of the aqueous extract of *Allium sativum* bulbs on laboratory mice and rats. CamJ Exp Biol.

[12] Behrens B, Karber G (1983). Mathematics for naturalists and agriculturalists.

[13] Gornall AG, Bardawill CJ, Maxima D (1949). Determination of serum protein by means of the Biuret reaction. JBiochem.

[14] Newman D, Prince C (1999). Renal function and nitrogen metabolites. Textbook of clinical chemistry.

[15] Reitman S, Frankel S (1957). A colorimetric method for the determination of serum glutamate, oxaloacetate and pyruvate transaminases. Am J Clin Path.

[16] Bergmeyer HU (1972). Standardization of enzyme assays. Clin Chem.

[17] Benson JP, Cales B: *Animal anat*omy *and physiology. Laboratory text book*. W.m.c. Brown Communication Dubuque, 1992.

[18] Mariano SH, Fiore DI (1963). An atlas of human histology.

[19] Kengni F, Tala DS, Djimeli MN, Fodouop SPC, Kodjio N, Magnifouet HN (2013). In vitro antimicrobial activity of *Harungana madagascriensis* and *Euphorbia prostrata* extracts against some pathogenic *Salmonella* sp. Int JBiolChem Sci.

[20] Nguelefack TB, Fotio AD, Watcho P, Wansi SL, Dimo T, Kamanyi A (2004). Analgesic properties of the aqueous and ethanol extracts of the leaves of *Kalanchoe crenata* (Crussaceae). Phytother Res.

[21] Eseinhauer L, Nichols WL, Spencer TR, Bergan WF (1998). Clinical pharmacology and nursing management Philadelphia.

[22] Njateng GSS, Kuate JR, Gatsing D, Tamokou JD, Mouokeu RS, Kuete V (2010). Antidermatophytic and dermal toxicity of essential oil from the leaves of *Ageratum houstonianum* (Asteracae). JBiol Sci.

[23] Panter KE, James LF (1990). Natural plant toxicants in milk. A review. J Anim Sci.

[24] Delongeas J, Burnel D, Netter P, Grignon M, Mor J, Royer R (1983). Toxicité et Pharmacocinétique de l’oxychlorure de zirconium chez les rats. Aust J Pharm.

[25] Shah AS, Wakade AS, Juvekar AR (2008). Immunomodulatory activity of methanolic extract of *Murraya koenigii* (L) Spreng leaves. Indian J Exp Biol.

[26] Aliyu R, Adebayo AH, Gatsing D, Garba H (2007). The effet of ethanolic leaves extract of *Commiphora Afrcana* (Burseraceae) on rat liver and kidney functions. JPharmacol Toxicol.

[27] Yagi SM, Tigani ELS, Adams SEI (1998). Toxicity of *Senna obtusifolia* fresh and fermented leaves, *Senna alata* leaves and some products from *Senna alata* in rats. Phytother Res.

[28] Ganong FW: Physiologie Médicale, Américaine Boerk, Université*.* 2002.

[29] Gome MB, Kouakou K, Toure A, Traore F (2011). Étude de la toxicité aiguë et subchronique de l’extrait aqueux de *Passiflora foetida* Linn. (Passifloraceae) chez les rats et les souris. Int J Biol Chem Sci.

[30] Azza Z, Marnissi F, Naya A, Benjelloun N, Zamyati S, Amrani M (2012). Toxicological evaluation of *Thymelaea hirsuta* and protective effect against CCl4-induced hepatic injury in rats. Int J Biol Chem Sci.

[31] Costa-Silva JH, Lima CR, EJR S, Arojo AV, MCCA F, Ribero RA (2008). Acute and sub-acute toxicity of the *Carapa guianensis*Aublet (Meliaceae) seed oil. J Ethnopharmacol.

[32] Taziebou LC, Etoa FX, Nkegoum B, Pieme CA, Dzeufiet DPD (2007). Acute and subacute toxicity of *Aspilla africana* leaves. Afric J Trad Med.

[33] Magnifouet NH, Ngono NAA, Kuiate JR, Koanga MMML, Tamokou JD, Ndifor F (2011). Acute and sub-acute toxicity of the methanolic extract of *Pteleopsis hylendron* stem bark. J Ethnopharmacol.

[34] Loh AH, Cohen AH (2009). Drug induced kidney diseases. Pathology and current concepts. Annals Acad Med.

